# 

**Published:** 1894-10-13

**Authors:** 


CONTENTS.

**Criminal Responsibility in the Insane** - 19

**Injuries of Joints** - 20

**On Toxic Neutralisations.**—By Sir Benjamin Ward Richardson, M.D., F.R.S.—II. Reconstitution - 21

**Medical Progress and Hospital Clinics:**

Notes on the Methods of Performing Paracentesis of the various Cavities—(*continued*) - 25

Progress in Medicine.—Anæsthetics—(*continued*) - 26

Progress in Surgery.—Abdominal Surgery - 28

New Drugs and Preparations - 30

New Appliances and Things Medical - 30

**The Psychologist in History.**—IV. The Kings of Spain - 22

**Annotations.**—The Japanese as Experimental Physiologists—Ireland for a Holiday—Death of Dr. Wendell Holmes - 23

**Notes and News** - 24

**The Institutional Workshop:**

Hospital Finance.—XI. Domestic Economy - 31

Hospital Sunday—Who Were its Founders and Originators? - 32

The Story of the Insane from Year to Year - 33

Around the Hospitals - 34

**Scraps and Gleanings** - 34

**The Book World of Medicine and Science** - 35

EXTRA SUPPLEMENT.—THE NURSING MIRROR.

**News from the Nursing World.**—Royalty at Leeds—Kindly Acknowledgment—Trained Nurses' Club—Thoughtless Critics—Sectarianism in Workhouses—Winter Workers Wanted—Royal National Pension Fund—Certificated but not Trained—An Increase of Staff—Progress at Exeter—Classes at Manchester—Welsh Nurses—The Nourishment of French Babies—Queen's Nurses in Ireland—Short Items - xii
**Notes for Nurses on Antiseptic Surgery.**—By William Horrocks, M.D., F.R.C.S.—I. The Development of Antiseptic Methods - xiii
**New South Wales** - xiv
**The Countess of Dufferin's Fund** - xiv
**Everybody's Opinion—Where to Go** - xv
**Dress and Uniforms.**—III. Indoor - xvi

